# New Strategies in Production and Product Quality Control of Fresh Meat

**DOI:** 10.3390/foods11203293

**Published:** 2022-10-21

**Authors:** Manuel Juárez

**Affiliations:** Agriculture and Agri-Food Canada, Lacombe Research and Development Centre, Lacombe, AB T4L 1W1, Canada; manuel.juarez@agr.gc.ca; Tel.: +1-403-782-8118

Meat consumption continues to increase worldwide, especially in developing countries, and this trend is expected to persist in the future, as the per capita income of the countries continues to grow. As a consequence, the meat trade landscape has become more complex in recent years, seeing an expansion in export/import volumes and values, as well in commodity and niche markets. Meeting the demands of both domestic and international buyers has created new challenges in the sector. Hence, quality assurance and classification systems are more important than ever, as buyers and consumers are becoming more exigent, and competition for the global market requires novel technologies able to meet such requirements. Moreover, concerns have been raised in developed countries regarding the potential negative effects of meat consumption on human health, the impact of livestock production on climate change, and the welfare of livestock. New recommendations from national and international health organizations suggest decreasing the consumption of red meats. Similarly, greater emphasis is being placed on producing livestock in a sustainable and humane manner. As part of the solution, producers and packers collaborate with researchers to provide potential strategies to address such complex challenges.

In this Special Issue of *Foods*, “New Strategies in Production and Product Quality Control of Fresh Meat”, we feel proud to have compiled a series of research manuscripts exploring new strategies to be implemented in the meat sector, both at the production and quality control stages. As expected for a topic of this magnitude, the research questions are diverse, including studies from animal genetics to meat flavour. Readers will find novel information regarding genes related to intramuscular fat in pigs [1], and the impact of production factors on beef primal composition [2], as well as microbiological studies evaluating the risks of *Salmonella* contamination in both chicken [3] and buffalo [4] meat. Two new systems to evaluate pork quality at the slaughter plant [5,6] are also presented in this issue, with emphasis on their potential adoption by the pork sector. Finally, two more studies evaluating physico-chemical and flavour traits in dry-aged beef [7] and traditional Chinese bacon [8] reflect on the idea that the whole production system is aimed at providing a satisfactory eating experience for the consumer. The quality of the manuscripts compiled in this Special Issue, submitted by internationally recognized research teams, will provide readers with valuable insight into the new trends that will be applied in the meat sector in the near future. For this reason, I would like to thank all authors for their contributions and professionalism during the review process. It has been my pleasure to act as Guess Editor and work closely with the team at *Foods*, especially Ms. Dana Min, Section Managing Editor.
